# Strategies to Preserve Postharvest Quality of Horticultural Crops and Superficial Scald Control: From Diphenylamine Antioxidant Usage to More Recent Approaches

**DOI:** 10.3390/antiox9040356

**Published:** 2020-04-24

**Authors:** Cindy Dias, Ana L. Amaro, Ângelo C. Salvador, Armando J. D. Silvestre, Sílvia M. Rocha, Nélson Isidoro, Manuela Pintado

**Affiliations:** 1 CBQF—Centro de Biotecnologia e Química Fina–Laboratório Associado, Escola Superior de Biotecnologia, Universidade Católica Portuguesa, Rua Diogo Botelho, 1327, 4169-005 Porto, Portugal; cdias@porto.ucp.pt (C.D.); aamaro@porto.ucp.pt (A.L.A.); 2 CICECO, Departamento de Química, Universidade de Aveiro, 3810-193 Aveiro, Portugal; angelomcsalvador@gmail.com; 3 LAQV-REQUIMTE, Departamento de Química, Universidade de Aveiro, 3810-193 Aveiro, Portugal; smrocha@ua.pt; 4 Cooperativa Agrícola dos Fruticultores do Cadaval, CRL (COOPVAL), Estrada Nacional 115, Km 26, 2550-108 Cadaval, Portugal; nelson.isidoro@coopval.com

**Keywords:** postharvest treatments, diphenylamine, superficial scald, physiological disorders control

## Abstract

Horticultural crops are vulnerable to several disorders, which affect their physiological and organoleptic quality. For about forty years, the control of physiological disorders (such as superficial scald) in horticultural crops, particularly in fruit, was achieved through the application of the antioxidant diphenylamine (DPA), usually combined with controlled atmosphere (CA) conditions. However, identification of DPA residues and metabolites in treated fruits, associated with their toxicity, banned the use of this antioxidant in Europe. This triggered the urgent need for novel and, ideally, natural and sustainable alternatives, combined with adequate storage conditions to protect cultivars from harmful agents. This review systematizes the state-of-the-art DPA application on several fresh cultivars, such as apples, pears, and vegetables (potatoes, spinach, etc.), as well as the possible mechanisms of the action and effects of DPA, emphasizing its antioxidant properties. Alternative methods to DPA are also discussed, as well as respective effects and limitations. Recent research on scald development molecular pathways are highlighted to open new non-chemical strategies opportunities. This appraisal shows that most of the current solutions have not lead to satisfactory commercial results; thus, further research aimed to understand the mechanisms underlying postharvest disorders and to design sustainable and safe solutions to improve horticultural products storage is needed.

## 1. Introduction

Postharvest physiological disorders control is crucial for the sector since these problems can be responsible for up to 50% of postharvest losses, depending on the crop quality, harvest method, time of storage, and shelving conditions. The physiological and organoleptic quality of horticultural products can be affected by numerous problems during postharvest storage, which often require the use of agrochemicals to preserve quality and extend shelf life [1].

**Figure 1:**
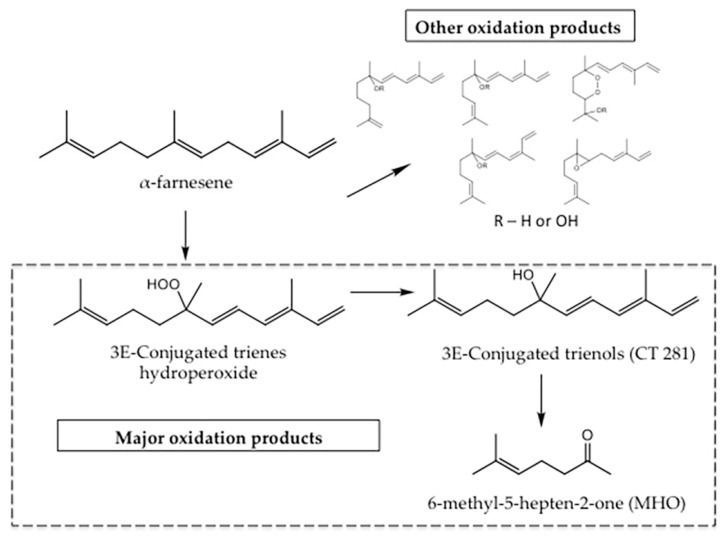
Schematic of α-farnesene auto-oxidation. Step represented by arrows indicates α-farnesene auto-oxidation into conjugated trienes, auto-oxidation into conjugated trienols and auto-oxidation into conjugated 6-methyl-5-hepten-2-one (MHO). R = OH represents conjugated triene and R = H represents conjugated trienols. 3E-Conjugated trienols (CT 281) (3E-7,11-trimethyl-1,3,5,10-dodecatetraene-7-ol) is one of the most abundant trienols resulting from α-farnesene auto-oxidation. Adapted from Lurie et al. [14].

Superficial scald is one of the most problematic postharvest problems, mostly associated with apple and pear skin disorders. It is the result of an oxidative process induced by cold stress, which can cause serious quality losses after long-term refrigerated storage, and is expressed as brown or black areas on the fruit peel [2,3,4]. Generally, the incidence of superficial scald depends on several factors, such as fruit cultivar [5,6], harvest time [7], and storage conditions [8]. This process is mainly triggered by the oxidation of α-farnesene, a secondary metabolite present in the pulp and skin of a wide range of fruits. This hydrocarbon sesquiterpene is chemically unstable and oxidizes easily, generating highly reactive conjugated trienols (CTs) (Figure 1) [9]. These CTs react with lipids and proteins affecting cell membranes integrity [10]; thus, allowing the contact between oxidative enzymes (i.e., polyphenol oxidase, PPO, and peroxidase, POX) and their respective substrates, which cause the typical fruit peel necrosis and off-flavors production [11,12,13]. Thus, it is widely accepted that the control of superficial scald disorder requires the maintenance of a good antioxidant system throughout storage, mainly to avoid α-farnesene oxidation.

**Figure 2:**
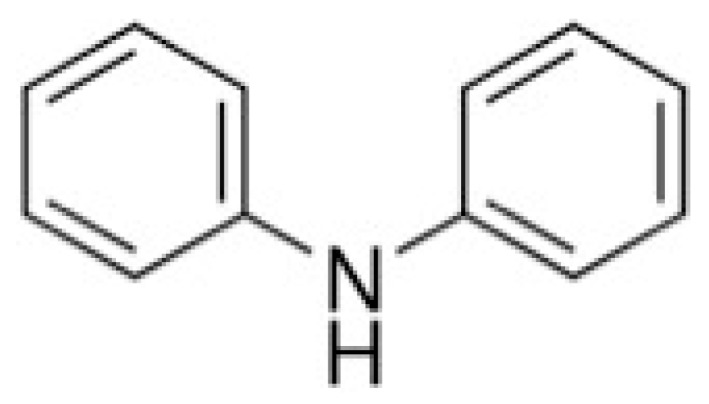
Chemical structure of diphenylamine (DPA).

For approximately 40 years, treatments that included chemicals, such as diphenylamine (DPA)—a liposoluble organic antioxidant (Figure 2)—were the main effective solutions used to control postharvest disorders (namely storage scald in fruits and vegetables), before it was banned in many countries [7,14].

Before the advent of the commercial use of DPA, the conventional method for superficial scald control was through the use of oil wraps around fruit, or ventilation inside storage containers, to reduce the concentration of α-farnesene in the fruit tissue [15]. However, it was shown that DPA was able to control scald more effectively than these traditional practices [16]. These findings led to the approval of the commercial use of DPA (dips or drenches), typically at 1 to 2 g L^−1^ in the early 1960s. Due to its low cost, simple application, and presumed low toxicity, it rapidly spread to many other countries [17,18,19,20].

Although superficial scald was considered to be effectively controlled using DPA, the detection of toxic and carcinogenic residues and metabolites of this compound in horticultural crops generated insecurity about its use. Besides, the rising alarms about the effects of synthetic chemicals on human health and the environment generated hesitation in the long-term future use of DPA [21,22,23]. Consequently, its use has been banned by the European Commission, all existing authorizations for the application of plant protection products containing DPA have been withdrawn, and DPA can only be present in products at the detectable Maximum Residue Limit (MRL) of 0.1 mg kg^−1^ [24]. This ban challenged the scientific community to search for novel non-chemicals, such as low-oxygen and dynamic atmosphere treatments for long-term storage [25]. For example, the prohibition of DPA use led the ‘Rocha’ pear sector to face significant product losses (about 15 to 30%).

The ‘Rocha’ pear is a Portuguese pear variety, with Protected Designation of Origin (PDO), representing about 93% of the national production of pear. The DPA use restriction is quite serious given that, without this antioxidant, the conservation of ‘Rocha’ pears and some varieties of apples produced in Portugal are reduced to three months, after which, symptoms of superficial scald become evident [25,26]. Whereas, with the use of DPA, they could be stored for up to 9 months. As a result, postharvest research has been devoting strong efforts to the development of safe and sustainable treatments for long-term storage.

Despite the demonstrated effectiveness of DPA, it is important to note that the occurrence of superficial scald is associated with several factors, including the sensitivity of the cultivar, harvest time and temperature, and O_2_ and CO_2_ availability (storage conditions).

To address these challenges, the present review is structured in three parts. First, the state-of-the-art DPA applications are discussed in detail, namely their effect on the horticultural varieties. Secondly, the reasons for DPA prohibition and studies supporting its toxicity are presented. The positive effects of the alternatives already used to mitigate the postharvest disorders, as well as their main limitations, are also presented. This knowledge will shed light on future research on postharvest disorder control solutions.

## 2. DPA in Superficial Scald Control

**Figure 3:**
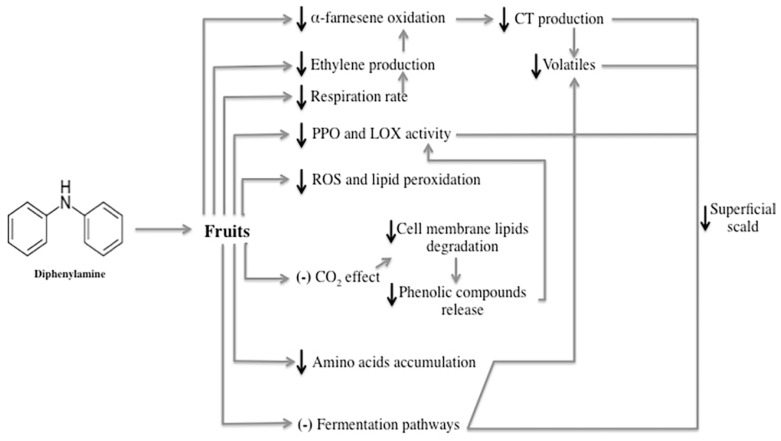
Proposed mechanisms of action of DPA on superficial scald control.

DPA has been widely used to prevent the postharvest deterioration of apples and pears and vegetable crops, namely from superficial scald. Its anti-scald activity is directly related to antioxidant properties, which protect the horticultural products from oxidation during storage [27]. The mechanisms of action of DPA in reducing superficial scald are not yet fully understood, and are inconsistent among different authors [14]. Although, the most widespread hypothesis is that DPA acts as an antioxidant and inhibits the oxidation of α-farnesene to CTs [27,28]. Additionally, other mechanisms on fruit metabolism are reported (as detailed below and represented in Figure 3), including reducing the respiration rate and ethylene production, extending maturation and senescence, and decreasing lipoxygenase (LOX) and polyphenol oxidase (PPO) activities [29,30].

In addition to the control of superficial scald, DPA also reduces the incidence of internal diseases during controlled atmosphere (CA) storage and maintains the physicochemical fruit quality, such as firmness, soluble solids content, and acidity [6]. Besides these unique features, other beneficial properties are attributed to DPA, such as its antifungal [31] and plant regulator properties [24].

Given the importance of the use of DPA gathered in postharvest treatments, its application was protected by many patents, some of which are worth mentioning. Notably, patent US3526518 [32], in which in 1967, the need for a liquid formulation of DPA suitable to prepare dispersions and emulsion in water (which could be used to prevent storage scald) was disclosed. In patent CA2277853A1 [33], Sardo presented a postharvest treatment that would extend the shelf life of fruits and vegetables while preserving normal ripening. The procedure involved the application of an aqueous composition with DPA as an antioxidant.

Other patented applications of DPA comprise its preservative effects on plants (patent US6129927A) [34], and its antagonistic activity of different strains from the blue mold in apples (US5525132A) [35].

A combination of compounds containing DPA was patented in 2004 as an effective controlling solution for phytopathogenic diseases (US 0265267A1) [31].

In 2008, Wartanessian filed US patent no. 0103212A1, where a procedure to apply solid DPA to fruits via aerosol application was disclosed [36].

A chronological summary of the horticultural, physiological, and biochemical effects of DPA reported in the literature is explained next and is presented in Table 1, with the indication of application methods and DPA concentrations, and is discussed in detail in coming sections.

### 2.1. Effect of DPA on Antioxidant Activity

Several studies have reported the effect of DPA on the inhibition of α-farnesene oxidation in vivo [28,29,43]. A strong relationship between CT 281 and scald has been often shown [8,26]. Huelin and Coggiola [8] demonstrated that postharvest application of DPA increased the total antioxidant content in apples and reduced the accumulation of the CTs, namely CT 269 and CT 281. This observation highlighted the DPA antioxidant activity against α-farnesene oxidation and, therefore, the reduced accumulation of CTs [8].

Furthermore, Anet and Coggiola [26] evaluated the ability of a variety of lipophilic antioxidants to control the auto-oxidation of α-farnesene. All groups of antioxidants tested, inhibited the α-farnesene autoxidation in vitro, but only DPA effectively avoided the accumulation of CTs in vivo [26]. These results prompted the investigation into how DPA acts in the prevention of superficial scald, in addition to the inhibition of α-farnesene auto-oxidation. Concerning this, some researchers have presented results demonstrating the reduction of α-farnesene biosynthesis in fruit preserved with DPA (see Table 1) [28,49].

In 1993, Sugihara et al. [58], studied the antioxidant potential of DPA, comparing its antioxidant ability with that of butylated hydroxytoluene (BHT)—one of the strongest antioxidants, and often used as reference—in inhibiting membrane peroxidation and reducing the formation of malondialdehyde, a compound associated with oxidative stress. BHT totally inhibited the formation of malondialdehyde, and DPA was only 10% less effective than BHT in inhibiting malondialdehyde formation, validating its antioxidant potential [58]. In this study, it was also reported that the antioxidant effect of DPA derives from the secondary amine group and its reactivity with the peroxyl radical [58]. Zhao et al. [49] also demonstrated that the application of DPA on ‘YaLi’ pears avoided the production of reactive oxygen species (ROS) and lipid peroxidation. Piretti et al. [40] obtained similar results with ‘Granny Smith’ apples. Their results showed the positive influence of 2 g L^−1^ DPA solution in the reduction of CTs production, which resulted in a 20% reduction of scald index [40]. In 2005, Arquiza et al. [43] tested a method of controlling the level of α-farnesene oxidation, which proved to be effective; it consisted in the application of 2 g L^−1^ DPA for 1 min to apples, followed by storage at 0.5 °C. This method made possible to control of α-farnesene oxidation [43]. Isidoro and Almeida [25] applied a postharvest drench of DPA (0.636 g L^−1^) to ‘Rocha’ pears, followed by storage at 0 °C and 2.5 kN m^2^ O_2_ + 0.7 kN m^2^ CO_2_. This treatment improved CTs scavenging and reduced scald by 53% [25].

### 2.2. Effect of DPA on the Enzymatic Activity

In addition to its antioxidant activity, the effect of a postharvest DPA treatment on the activity of some of the oxidative enzymes involved in superficial scald disorder was studied by Lurie et al. [30]. After six months of storage, the PPO activity in apple peel tissue treated with DPA was 45% lower than that found in untreated apples. These authors also concluded that in the peel areas where scald developed, PPO activity was three times higher than in healthy peels. The affected peel had increased PPO activity after one week at 20 °C. The peroxidase activity (POX) in fruit treated with DPA was 30% lower than in untreated fruit. Interestingly, in contrast to the increased PPO activity found in scald tissue, POX activity in tissue showing scald was four times lower than in healthy tissue. Regarding lipoxygenase activity, it was 50% lower in apple peel treated with DPA but higher in untreated apple peel [30].

The study of isolated apple, potato, and turnip mitochondria also showed that cytochrome oxidase was not affected by DPA, but the succinoxidase system and nicotinamide adenine dinucleotide (NADH) oxidase, as it is related to membrane integrity, was inhibited [37,53].

In a review, Toivonen and Brummell [59] cited several studies showing that DPA counteracts the inhibitory effect of CO_2_ on fruit stored under controlled atmosphere (CA). They hypothesized that this effect contributed to the protection against degradation of cell membrane lipids, inhibiting the release of phenolic substrates to the cytosol, where polymerization catalyzed by the enzyme PPO occurs, and consequently leads to scald appearance [59]. Carrasco-Rodriguez et al. [57] also evidenced the inhibitory effect of DPA on glutathione reductase and POX activity, and Yihui et al. [47] demonstrated that the application of a DPA formulation commercially available as Decco No Scald^®^ DPA AEROSOL reduced laccase activity.

### 2.3. Effect of DPA on the Respiratory Rate

Sims [52] found that DPA decreased the respiratory rate in both the apple cortex and peel tissue (see Table 1). Some works also indicate that DPA reduces plant cell respiration by inhibiting the transport of mitochondrial electrons [53,56]. Baker [53] demonstrated that DPA delayed the reduction of mitochondrial cytochrome in the sweet potato succinoxidade system; thus, affecting the electron transport chain. Oettmeier and Renger [55] also reported the inhibition of photosynthetic electron transport and photophosphorylation by DPA on spinach chloroplast. Janisiewicz et al. [44] associated the effect of DPA on ‘Golden Delicious’ apple respiration with the decrease of blue mold disease.

### 2.4. Effect of DPA on Ethylene Biosynthesis

Ethylene is a plant growth regulator that affects ripening, senescence, and quality of fruits [29]. Ethylene diffuses into the cell’s endoplasmic reticulum binding to specific receptors, initiating maturation process, and determining global quality. There is evidence that ethylene promotes α-farnesene synthesis [4,29]. Du and Bramlage [29] found that DPA at 2 g L^−1^ reduced ethylene production and, consequently, the synthesis and oxidation of α-farnesene, with diminished surface scald in ‘Cortland’ apples. In general, cumulative data sustain the hypothesis that ethylene stimulates α-farnesene synthesis, possibly stimulating transcription and translation of 3-hydroxy-3-methylglutaryl-CoA reductase, and/or α-farnesene synthase genes. Furthermore, it appears that the reduction in α-farnesene synthesis caused by high levels of DPA and ventilated CA storage is, at least in part, due to the decrease in ethylene production [29].

In a study by Lurie et al. [30], ethylene production by apples treated with DPA, during three months of storage, was lower than that of untreated apples, which means reduced ripening, as it is known that ethylene production induces fruit maturation. Moreover, Lurie et al. [30] reported that treatment with DPA slowed down ripening and senescence after prolonged storage. Thus, these authors concluded the positive effect of DPA on increasing the storage life of fruit.

Fruit softening and loss of acidity are two important indicators of fruit ripening and senescence and are related to increased ethylene production. Hardenburg and Anderson [60] reported that different varieties of apple treatment with 1 or 2 g L^−1^ of DPA had no negative effect on the firmness and chemical composition of the fruit, validating the lower ethylene production with DPA treatment.

### 2.5. Effect of DPA on Amino Acids

DPA also influenced the accumulation of total amino acids, in DPA treated-apples [61]. Postharvest literature in this field demonstrated that, when symptoms of superficial scald were high, the level of alanine, valine, leucine, serine, aspartic acid, methionine, cysteine, and phenylalanine in the fruit were also high, but lower in DPA-treated apples [48]. Moreover, scald has been associated with high γ-aminobutyric acid (GABA) levels in ‘Honeycrisp’ apples [62]. More recently, Karagiannis et al. [48] observed an extensive down-accumulation of several proteins following DPA treatment, verifying the importance of protein repression studies to scald prevention, namely cell membrane protection. Their closed global protein analysis showed that sulfur amino acids are possibly involved in scald [48]. The high levels of some amino acids may be related with high quantities of volatile metabolites resulting from amino acids, as some are direct or indirect substrates for the biosynthesis of volatile compounds such as alcohols, carbonyls, and esters [63].

### 2.6. Effect of DPA on Volatile Compounds

In 1919, volatile compounds, namely esters and terpenes, produced by apples during low temperature storage were suggested to be indicators of surface scald development by Brooks et al. [64]. During low-temperature storage, volatile compounds such as α-farnesene and its oxidation products (CTols) are continuously produced [8,65]. These compounds are retained in the fruit cuticle and are accumulated at high levels in affected fruit [42].

The production of esters derived from 1-hexanol in fruit such as apple and pear tends to increase after harvesting and then decreases during long-term storage. However, the production of esters in fruit affected by scald was reduced by about 50% with the application of DPA [42]. The production of esters derived from 1-hexanol was further reduced in the development of scald, with an increase of hexyl-2-methylbutanoate. The production of methyl butanoate was detected only in scalded fruits. Besides, the primary volatile oxidation product of α-farnesene, 6-methyl-5-hepten-2-one (MHO), was present only in fruits with scald. The reported data suggested that the inhibition of esters production may occur as a result of the physiological changes associated with the susceptibility to scald [42].

In a metabolomic study, Lee et al. [61] found that volatile compounds such as acetaldehyde, ethanol, ethyl esters, 1-propanol, 1-butanol, and 2-methyl-butanol are strongly associated with the development of disease symptoms in untreated apples. These researchers found a strong difference between the concentrations of these volatile compounds, produced in the control fruit (without treatment) and in fruit treated with DPA. The fact that acetaldehyde and ethanol are found at lower levels in fruit treated with DPA shows that the fermentation pathways are suppressed [61]. Consequently, ethyl-esters production is also reduced because it is catalyzed by the alcohol acyl CoA transferase which requires the presence of ethanol [61]. Brooks et al. [64] treated ‘Delicious’ and ‘Stayman Winesap’ apples with 1 g L^−1^ of DPA and 5% isopropyl alcohol and no changes were observed in the volatile profile, which justified the scald control [64].

Another important factor that associates the production of specific volatile compounds to the symptoms of superficial scald is the biosynthesis of fatty acids and the formation of mono- and diterpenes precursors. The formation of these compounds occurs in the chloroplasts, which are affected by superficial scald, reinforcing the principle that changes in volatile emissions may accompany the onset of symptoms [32].

### 2.7. Other Postharvest Diphenylamine Effects

DPA effects were also evaluated on vegetable crops, although not as frequently. In a work performed by Gilbert et al. [54], the foliar spray with 1% DPA reduced the O_3_ injury by 50% in bean, petunia and tobacco crops. Moreover, Purvis et al. [56] revealed that the DPA application up to 12 mmol L^−1^ reduced the chilling-induced pitting of green bell pepper (*Capsicum annuum* L.) fruit. The treatment was effective whether applied as a 2 min dip or injected into the seed cavity before storing peppers at 1 °C for 6 or 8 d, followed by storage at 20 °C for 2 d [56]. Paliyath and Guelph [66] claimed the use of DPA in combination with other components to improve the quality and shelf life of fruits and vegetables, partially processed, such as apples.

DPA was effective to avoid phytopathogenic contamination, especially against Ascomycetes, Basidiomycetes, Fungi imperfecti, and Oomycetes, in leguminous plants, such as beans, lentils, peas, or soybeans; oil plants, such as mustard, poppy, olives, sunflowers, coconut, castor oil plants, cocoa beans, or groundnuts; melon plants, such as pumpkins, cucumbers, or melons; and fiber plants, such as cotton, flax, hemp, or jute [31]. Moreover, DPA was used to prevent oleocellosis in Valencia oranges by regulating malondialdehyde (MDA) levels and antioxidant enzyme activity [51].

## 3. Toxicity of DPA Supporting EU Prohibition

Despite its widespread use until 2014, ecotoxicological findings showed that DPA is acutely toxic to rabbits, mice, rats, and many other aquatic species [21,22,23,67]. Exposure generally takes place through skin, ingestion, or inhalation; the targets are blood, kidney, and the liver [67], and it leads to dermatitis effects (vesicular and exudative eczemas) [68,69,70]. DPA was also shown to trigger inhibitory effects on the photosynthesis of phototrophic bacteria. Some authors reported the effect of DPA on the inhibition of the carotenoid synthesis in some photosynthetic bacteria, such as *Rhodopseudomonas sphaeroides* [71,72].

According to the National Institute for Occupational Safety and Health (NIOSH), as well as other studies, DPA can generate potential carcinogens and mutagenic compounds, such as sulphonyl and glucuronyl conjugates, and nitrosamines [21,24,73]. Apples were tested for the presence of DPA residues, and the United States department of Agriculture (USDA) found that about 20% had DPA residue above the U.S. Environmental Protection Agency (EPA) tolerance level of 10 mg kg^−1^ [74]. Moreover, in a study concerning DPA degradation in immersed apples, it was shown that DPA residues infiltrate from the surface into the pulp. After 40 weeks of storage, the apples still presented some presence of residue [24]. For these reasons, the European Commission has banned its use in 2014 and all existing authorizations for its application to plant protection. Since studies about the biodegradability and environmental fate of DPA are scarce, further research is required to determine the complete dimension of its potential environmental impact and to introduce possible (bio) remediation techniques for locations that are contaminated.

## 4. Alternatives to DPA in Controlling Postharvest Quality and Superficial Scald

In line with preceding information, the increased health concerns from consumers, as well as the pressure from export markets regarding postharvest chemical treatments [75] led to the restriction on the use of DPA to prevent the incidence of physiological disorders in vegetables and fruit. These issues and the growing demand for safer fruit and vegetables warrant the investigation into alternative strategies to control horticultural disorders. In fact, the search for new alternatives started even before the DPA prohibition, with the objective of replacing the use of synthetic food additives. Table 2 lists the alternatives developed over the years to the use of DPA and the drawbacks.

Even before DPA commercial approval, one of the first approaches to control surface scalding was the wrapping of apples in paper infiltrated with oils (see Table 2), such as mineral, olive, peanut [64,76], or castor [107] oils. The hypothesis was that oils had high gases absorbing power providing control of scald [64]. Prompted by these results, more recent studies reported the effects of other plant-derived oils [75,84,104]. Isolated components, namely mono-, di- and triacylglycerols lipids and phospholipids from vegetable oils such as soybean, corn, peanut, linseed, and olive oil, were shown to be effective on reducing the scald on ‘Delicious’ apples [84]. Similarly, Ju and Curry [75] found that stripped corn oil emulsions reduced superficial scald as effectively as DPA after 6 months of air storage, but became less effective after 8 months.

As early as 1935, Kidd and West [108] warmed the ‘Newton Wonder’ apple every 2 or 4 weeks and observed scald inhibition. Likewise, weekly warming-controlled scald in ‘Cortland’, ‘Delicious’, and ‘Law Rome’ apples [77]. Smith [109] proved that warming of 5 d at 15.5 °C or 3–5 d at 20 °C also controlled scald in apples. However, the reported results demonstrated that warming treatment is cultivar dependent.

Another practice before DPA replacement was the use of ventilation [17,64]. Brooks et al. [64] achieved a considerable reduction of scald by increased ventilation, suggesting that apple scald is due to the accumulation of volatile or gaseous substances on the surrounding environment that apple produces during metabolism. Huelin and Coggiola [8] found that increasing ventilation rates by a factor of 10 decreases scald incidence because it promotes α-farnesene evaporation and, therefore, avoids oxidation and consequent formation of CTs. However, some contradictory results, in which this practice leads to an increment on α-farnesene production, were also reposted [8].

In 1973, Gough et al. [78] tried to reduce superficial scald on ‘Cortland’, ‘Delicious’, and ‘Granny Smith’ apples applying one of the most potent commercially available antioxidants, namely BHT. This approach was as effective as DPA, albeit at higher concentrations [78]. More recently, the application of 10 mg L^−1^ of the natural antioxidant resveratrol and further storage at 1 °C inhibited scald appearance in 88% but was not as effective as DPA [91].

In 1974, Wills and Scott [79] tried to support the relation between minerals and scald development. A method consisting of EDTA application was used since EDTA forms stable chelates with several minerals and may contribute to remove these minerals from fruit. Injection of low concentrations of EDTA (<0.5 µmoL per fruit) into the core of ‘Jonathan’ apples, reduced superficial scald because of the strong EDTA chelating effect with cobalt and cooper minerals. These minerals were tested separately and promoted the occurrence of scald. However, at higher concentrations (>1 µmoL per fruit) EDTA increased the disorder because it inactivates minerals, namely barium and strontium. Barium and strontium were added to the fruit and decreased the incidence of scald [79].

Other treatments using coatings, which include waxes, lecithin, formulations based on sucrose esters and chitosan coatings, have already been reported in the control of superficial scald [104,107,110]. Moreover, the application of sucrose ester-based coatings (commercially available as Prolong and Nutri Save) reduced scald in ‘Bartlett’ and ‘d’Anjou’ pears but affected the natural ripening of the fruit [111]. Additionally, Sumnu and Bayindirli [98] investigated the influence of edible sucrose polyester-based and carnauba wax and shellac-based coatings (commercially available as Semperfresh^TM^ and Jonhfresh^TM^ respectively) on the postharvest quality of ‘Ankara’ pears. Both coatings were effective in extending the pears postharvest life and delaying ripening. Chellew and Little [80] achieved similar results using Semperfresh^TM^, which showed the capability to reduce scald for forty-three weeks when combined with controlled atmosphere (CA) conditions (see Table 2). Chitosan coatings also inhibit the development of scald; their efficacy is associated with an increase in chitosan concentration, but it declines for long storage periods [104].

Interestingly, the Israeli company Decco–Safepack Products Ltd., has presented a product line in the category of storage treatments, about which very little is known [112]. They propose that the solution to this problem will be the application of much lower concentrations of DPA than those previously used, combined with 1- methylcyclopropene (1-MCP) [112]. A Spanish company, Concentrol^®^, also emerged with an alternative for the shelf life extension of fruit called FRUPREVENT-50, which is an edible coating based on lecithin and other food additives, such as fatty-acids and sucrose, which creates a semipermeable membrane on the surface of the fruit. It can reduce scald and maintain the freshness of the fruit, extending its storage life [113]. However, despite the success demonstrated in the application of edible coatings, their efficacy is quite dependent on fruit cultivar, in opposition to DPA [113].

Vaporization with volatile compounds was also a solution tested by researchers. Sealed packages with vaporized ethanol controlled scald in ‘Granny Smith’ apples (see Table 2) [82,83]. However, they cause internal browning after two months of storage [114]. Vaporizations with propan-1-ol and butan-1-ol were as efficient as ethanol in the control of scald on ‘Delicious’ apples [114].

Hypobaric storage was introduced by Burg and Burg [115]. It involves storing of fruit with ventilated air at less than atmospheric pressure. Wang and Dilley [85] demonstrated that hypobaric stored ‘Granny Smith’ and ‘Law Rome’ apples did not develop superficial scald by application of 2 months of hypobaric storage plus, 6 months of CA.

Most recommendations for postharvest handling have focused on rapid cooling to reduce ripening and senescence processes [116]. Of interest to researchers in mitigating postharvest disorders include non-chemical treatments, such as CA storage, dynamic CA, and temperature conditioning—namely intermittent warming during storage, which can be as effective as conventional practices [117].

Controlled atmosphere (generally > 1% oxygen (*v*/*v*)) and low oxygen stress (generally < 1% oxygen (*v*/*v*)) are among the common treatments used, especially by the pome industry [118]. These strategies are known for reducing ethylene synthesis and respiration rate; thus, leading to superficial scald reduction [119]. In general, CA storage is associated with delayed scald. However, it is affected by cultivar and length of storage and its inefficacy to completely control postharvest disorders led to the rise of low-oxygen storage [14,118]. Storage under low oxygen was reported to control scald more effectively than standard CA [14].

Zoffoli et al. [97] tested different pre-storage treatments in conserving ‘Granny Smith’ apples commercial value. The use of temperature conditioning (10 d at 3 °C) and ultra-low oxygen 0.2–0.5 kPa achieved the best control of fruit hazards, compared to 1-MCP treatment. However, negative effects have been attributed to the use of low oxygen, such as core breakdown and high production of off-flavors in other fruit cultivars [118,120]. Exploring the different results obtained among researchers, Prange et al. [119] concluded that the oxygen level in storage should be just above the anaerobic compensation point (ACP), which can be inferred by the measurement of chlorophyll fluorescence, in order to reach the best benefit and avoid anaerobic undesirable effects. An improved and more recent version of CA, called dynamic CA (DCA), is receiving popularity among postharvest producers. It can be generally defined as a system that allows the customization of oxygen levels [118]. Weber et al. [94], Mditshwa et al. [95,96], and Wright et al. [121] are authors that focused on the effect of DCA to control physiological disorders (see Table 2). Besides the effort that has been made to transform DCA as a postharvest method, it should be noted the diversity of orchard practice and technological complexities among postharvest industries. This means that, while some markets are close to producers and distributors, others require long shipment periods [118]. For example, Mditshwa et al. [95] studied the potential of DCA on ‘Granny Smith’ apples over two growing seasons. Apples were stored in DCA at 0 °C for 5 d up to 20 weeks followed by 6 or 10 weeks simulated handling temperature (−0.5 °C) for long distant supply chains, plus 7 d at 20 °C [95]. Their results showed that DCA controlled superficial scald in both growing seasons after 6 weeks of shipping period. Yet, the threat of superficial scald development after 10 weeks was observed [95]. Additionally, CO_2_ injury expressed as internal browning or cavity formation is another protuberant illness in DCA [86,89]. The development of O_2_ and CO_2_ injuries both in CA and DCA continues a big fretfulness. Consequently, future research is needed to avoid such problems.

Another storage chemical treatment currently used to minimize superficial scald disorder is the application of 1-MCP, a widely registered inhibitor of ethylene synthesis [14]. This compound can reduce superficial scald by blocking ethylene receptors and thereby decreasing the accumulation and further oxidation of α-farnesene [14]. Although 1-MCP is generally accepted as effective, producers are reluctant to use it, because of the effects on maturation blockage and the consequent modification of fruit quality. So, the researchers also tried to evaluate the simultaneous application of 1-MCP and ethylene and other storage strategies [87] (see Table 2). Du et al. [93] demonstrated the influence of 1-MCP in the reduction of various chemical and physiological phenomena associated. Moreover, Milinkovic et al. [122], studied the impact of 1-MCP on five apple cultivars grown in Serbia, and concluded, that, besides 1-MCP effectiveness, it is important to take fruit maturity into account to select the appropriate moment for 1-MCP application.

Paliyath et al. [66] studied the influence of a postharvest spray containing three different solutions (solution A: 0.083 mol L^−1^ Hexanal; 0.057 mol L^−1^ Geraniol; 0.044 mol L^−1^ Geranyl Acetate; 0.030 mol L^−1^ Coumaric acid; 0.0088 mol L^−1^ Benzyl Adenine; solution B: 0.056 mol L^−1^ L-Ascorbic acid; 0.024 mol L^−1^ Ascorbyl palmitate; 0.015 mol L^−1^ C-tocopherol; 0.002 mol L^−1^ C-tocopherol acetate; solution C: CaCl_2_ 1% (*v*/*v*)) (see Table 2). They were able to reduce scald, whether the apples were stored in air or under in CA conditions [66]. No information about the potential mechanism behind scald reduction was revealed.

Phenolic fatty-acid esters from the peel of ‘Gala’ apples were reported as potential intrinsic anti-scald agents [123]. This approach is based on the role of phenolic compounds as postharvest physiological damage inhibitors, which results in the antioxidant activity associated with these compounds. In fact, it was already demonstrated that a higher concentration of antioxidants in the fruit decreases the susceptibility to scald [26,124].

Gatto et al. [106] tried the application of water solutions with CaCl_2_ and NaHCO_3_, which demonstrated a preservative effect, but not as successful as DPA.

More recently, insights into the molecular mechanisms of this treatment were achieved by Honaas et al. [117] by analysis of transcriptional responses of ‘Granny Smith’ fruit peel during early phases of long-term storage and after different warming treatments. The authors described a list of candidate genes, namely MDP0000265728, MDP0000164538, MDP0000170826, and MDP0000284624 involved in promotion as well as mitigation of this disorder [117]. According to the authors, this genetic traceability is potentially useful for the development of novel postharvest technology. Busatto et al. [125] performed an integrated metabolite screen in combination with a large-scale transcriptome study. They hypothesize that improved tolerance to low temperatures could be achieved by structural stabilization of the membranes through the production of unsaturated and long chain fatty acids. Moreover, genetic analysis performed by Ding et al. [103] revealed that CTs, ethylene production rate and programmed cell death are more associated with superficial scald than ROS.

This growing literature on molecular aspects of superficial scald, namely it causes, can be exploited to create new potential treatments.

## 5. Conclusions

The susceptibility of different fruits and vegetables to postharvest problems is a reality and depends on several factors: genetic, biochemical, and physiological. This review outlined an analysis of horticultural crops preservation, particularly focused on superficial scald protection after DPA prohibition. Because of its restriction, alternative strategies are being inspected regarding their potential in controlling postharvest crops. Among the alternatives, 1-MCP and controlled atmospheric conditions during storage are undoubtedly the current successful strategies to control postharvest quality, mainly through reducing ethylene synthesis and respiration rate. However, these have not achieved equivalent performance as DPA, so far. Moreover, negative outcomes, mainly cost and sensory quality constraints associated with these technologies, have been identified.

DPA has a set of effects on horticultural crops metabolism. In this respect, comprehensives studies reliant on DPA metabolism effects are essential to the profitable development of alternatives for superficial scald prevention and horticultural quality guarantee, with minimum impact on other physiological processes. Gathered results indicate that DPA foliar spray on vegetables and legumes acted as a remarkable crop protector from phytopathogenic contamination and chilling injury. Of great relevance is DPA’s potential against peel disorders on pome fruit. Typically, DPA dip at concentrations between 1–3 g L^−1^ inhibited either the occurrence of scald and/or chilling injury, permitting long-term storage. Therefore, it is important to keep in mind that DPA, in essence, is not only associated with its antioxidant protection, but also its synergic effects.

Knowledge about the different technologies already applied (and their limitations) will facilitate the research, development, and optimization of natural alternatives contributing to disordering mitigation. New studies are encouraged to clarify the biochemical basis of scald disorder, giving a high throughput to the development of adequate control strategies.

## Figures and Tables

**Table 1:** Diphenylamine (DPA) application and effects on fruits and vegetables.

Fruit and Vegetable Commodity	Application Method	Concentration and Treatment Conditions	DPA Effects/Important Facts	References
Apple	Mitochondrial isolation; Reaction of mitochondrial isolates with DPA	Not reported	Cytochrome oxidase not affected Succinoxidase system inhibition	[37]
‘Jonathan’ apple	Postharvest spray	Combined application of DPA and calcium	Reduction of bitter pit	[38]
‘Granny Smith’ and ‘Crofton’ apple	Postharvest coating	1–28 mg L^−1^	Reduction of α-farnesene oxidation Reduction of CT 269 and CT 281 production Increase of the total antioxidant concentration	[8]
Apple	Postharvest spray	5 g L^−1^ DPA; ethyl-hexanoate, 5 g L^−1^; and 45 g of a mixture of 9% (*v*/*v*) TritonX-35 and 91% (*v*/*v*) Triton X-102	93% of scald control	[32]
‘Jonathan’ apple	Postharvest injection into the core	DPA in ethanolic solution (0 to 2 mL)	DPA expected to be more effective in later storage	[39]
Different varieties of apples	Grower treatment	Concentration not revealed 1 °C	Inhibition of α-farnesene oxidation if the antioxidant content remained adequate	[26]
‘Granny Smith’ apple	Postharvest dip ~30 s	3 g L^−1^; 0 °C	Greater firmness and acidity Lower respiration rate Lower activities of PPO, POX and LOX Reduction of ethylene production	[30]
‘Cortland’ apples	Postharvest dip	2 g L^−1^	Reduction of ethylene production, synthesis and oxidation of α-farnesene Reduction of the incidence of surface scald	[29]
‘Granny Smith’ apple	Postharvest dip ~20 s	2 g L^−1^	Reduction of scald index by 80% Lower levels of CTs	[40]
‘Granny Smith’ apple	Postharvest dip	2.5 g L^−1^	No influence on internal fruit properties DPA more effective after 4 months than 6 months	[41]
‘Cortland’ apple	Postharvest dip ~30 s	997.1 mg L^−1^; 0 °C; RH > 95%	No influence of DPA on aroma compounds	[42]
Pome fruit	Postharvest dip	1 g L^−1^	DPA treatment influenced by the temperature	[5]
‘Empire’ apple	Postharvest drench	2 g L^−1^; 1 °C; 1.5% O_2_	Synergic effect between DPA and low O_2_ Delay of the onset of CTs production by ∼5 weeks and reduced CTs accumulation	[28]
‘Delicious Apples’	Postharvest immersion ~1 min	2 g L^−1^; 0.5 °C	A slight decrease in α-farnesene level Inhibition of CTs accumulation	[43]
‘Golden delicious’ apple	Postharvest spray	2 g L^−1^	Decrease of blue mold disease	[44]
‘Empire’ apple	Postharvest dip	0.3 to 1.2 g L^−1^	External CO_2_ injury inhibition even at low concentrations	[45]
‘Cortland’ and ‘Law Rome’ apple	Postharvest dip ~1 min	2 g L^−1^; 0.5 °C	Minimal delays between harvest and DPA are necessary to maximize control of scald Reduction of senescence breakdown	[46]
‘Cortland’ and ‘Schlect Spur Red Delicious’ apples	Postharvest vaporization	Decco No Scald^®^ DPA AEROSOL	Reduction of laccase activity	[47]
‘Granny Smith’ apple	Postharvest dip ~1 min	2 g L^−1^; 0 °C; 95% RH	DPA influence on total amino acids accumulation	[48]
‘Rocha’ Pear	Postharvest drench	636 mg L^−1^; 0 °C in air or in 2.5 kN m^2^ O_2_ + 0.7 kN m^2^ CO_2_	No effect on the α-farnesene level Improved CTs scavenging Reduction of scald index by 53%	[25]
‘YaLi’ pears	Not revealed	Not revealed	Removal of ROS and inhibition of membrane lipid peroxidation	[49]
‘Dangshansuli’ pear	Postharvest drench	1 to 2 g L^−1^	Reduction of α-farnesene, CTs, total phenols, PPO activity, and MDA	[50]
‘Valencia’ oranges	Postharvest dip ~3 min	1.183 g L^−1^	Control of oleocellosis by MDA	[51]
*Malus Sylvestris*	Postharvest spray	1 g L^−1^ or 2 g L^−1^	Decrease of glucose breakdown Decrease of CO_2_ and O_2_ uptake	[52]
Sweet potato *(Ipomoea batatas*) and turnips (*Brassica rapa*)	Mitochondrial isolation Reaction of mitochondrial isolates with DPA	≥ 0.163 g L^−1^	Succinoxidase and NAPH oxidase system inhibition Inhibition of electron transport	[53]
Bean, melon, petunia, and tobacco	Foliar spray	1%	Reduction of O_3_ damage by 50% or more	[54]
Spinach	Into the soil or spray the leaves	16.9 mg L^−1^	Inhibition of photosynthetic electron transport and photophosphorylation	[55]
*Capsicum annuum* L. (green bell pepper)	Postharvest dip ~2 min or injection into the seed cavity	2.028 g L^−1^	Reduction of chilling-induced pitting	[56]
Leguminous plants	Not revealed	Not revealed	DPA avoid phytopathogenic diseases	[31]
*Solanum tuberosum* cv. Agria (Potatoes plants)	Foliar spray	33.8 mg L^−1^	Increase of fresh weight production Reduction of glutathione, reductase, and guaiacol POX activity DPA protection against O_3_ injury	[57]

DPA = diphenylamine; CT = conjugated trienols; PPO = polyphenol oxidase; POX = peroxidase; LOX = lipoxygenase; ROS = reactive oxygen species; MDA = regulating malondialdehyde; RH = relative humidity.

**Table 2:** DPA alternatives on fruits and vegetables.

Fruit and Vegetable	Application Method	Treatment Conditions	Effects/Important Facts	References
‘Stayman Winesap’ and ‘Grimes’ apples	Postharvest wrap with oils	Mineral oil, olive oil, peanut oil	**Effects:** reduction of apple scald around 80% in some cases; greener and firmer fruit **Drawbacks:** off-flavors production, contrary to DPA application effect	[64,76]
‘Cortland’, ‘Delicious’ and ‘Law Rome’ apple	Intermittent warming	Intermittent warming to 20 °C for 24 h every 1,2 or 4 weeks during cold storage for 16 weeks	**Effects:** intermittent warming reduced scald **Drawbacks:** scald incidence variation among cultivars in opposition to DPA effectiveness in all cultivars	[77]
‘Granny Smith’ and ‘Crofton’ apple	Postharvest chambers ventilation	Increase of flow rate by a factor of 10	**Effects:** increased α-farnesene evaporation **Drawbacks:** α-farnesene production increase in some cultivars, which did not occur with DPA use	[8]
‘Cortland’, ‘Delicious’ and ‘Granny Smith’ apple	Postharvest dip	BHT at higher concentrations than DPA (i.e., >2 g L^−1^)	**Effects:** as effective as DPA **Drawbacks:** higher concentrations than DPA to achieve the same effect; non-natural treatment	[78]
‘Granny Smith’ apple	Postharvest injection	< 0.5 µmol of EDTA/ fruit	**Effects:** scald reduction **Drawbacks:** increased disorder at higher concentrations, contrary to DPA	[79]
‘Granny Smith’ apple	Postharvest dip	Storage atmosphere with low ethylene, Semperfresh^TM^ (0.5 g L^−1^), ascorbic acid (5 and 10 g L^−1^), ascorbyl palmitate (10 g L^−1^) and citric acid (3 g L^−1^).	**Effects:** reduction of scald incidence by Semperfresh and ascorbic acid in a controlled atmosphere for 43 weeks **Drawbacks:** no reduction of scald in low ethylene; no reduction of scald by Semperfresh^TM^ and ascorbic acid in air; none of the coating showed the performance of low concentration of DPA (0.5 g L^−1^)	[80]
‘Red Chief’ and ‘Golden Delicious’ apples	Postharvest dip	Semperfresh^TM^ at 1% combined with ascorbyl palmitate, and n-propyl gallate; 0 °C for 4 months	**Effects:** scald reduction after removal from cold storage **Drawbacks:** scald appearance after 10 days at room temperature differing from DPA effect, where no scald was observed	[81]
‘Granny Smith’ apple	Sealed bags with vaporized ethanol	0.5 and 1 kg kg^−1^ of ethanol/g fruit	**Effects:** scald reduction **Drawbacks:** browning induction after 2 months, contrasting to DPA browning inhibition	[82]
‘Granny Smith’ apple	Postharvest packaging	Ethanol, propanol, butanol, pentanol, hexanol at 0.04, 0.08 and 0.16 mol kg^−1^	**Effects:** diminution of the incidence of scald **Drawbacks:** undesirable side effects in the apple with butanol, pentanol, hexanol; thus, not as effective as the conventional application of DPA	[83]
‘Delicious’ apples	Postharvest dip ~2 min	6% and 9% (*w*/*v*) neutral lipids (mono-, di-, and triacylglycerols) or phospholipids from plant oils	**Effects:** reduction of scald after 6 months of cold storage **Drawbacks:** not as effective as 2 g L^−1^ of DPA	[84]
‘Granny Smith’ and ‘Law Rome’ apple	Postharvest atmosphere	Hypobaric storage at 5 kPa	**Effects:** superficial scald inhibition after 2 months of hypobaric storage plus 6 months of CA **Drawbacks:** not industrially feasible as DPA	[85]
‘Granny Smith’ apple	Postharvest atmosphere	DCA at 0.8 °C and 95% RH complemented with a non-destructive monitoring system for low oxygen stress of chlorophyll-containing fruit	**Effects:** optimization of low-oxygen storage; after 6 months no scald development; no influence of low-oxygen on taste and firmness **Drawbacks:** not profitable as DPA use	[86]
‘Empire’ apple	Postharvest drench	1-MCP at 1 µL L^−1^ for 24 h and 0 °C	**Effects:** reduction of superficial scald **Drawbacks:** non-natural treatment; blockage of maturation process contrary to DPA effect	[87]
‘Granny Smith’ apple and ‘Beurre d’Anjou’ pears	Postharvest dip ~3 min	2.5, 5 and 10% (*v*/*v*) corn oil emulsion; 0 °C	**Effects:** reduction of ethylene and α -farnesene production; reduction of scald in both apples and pears **Drawbacks:** not as effective as 2 g L^−1^ of DPA after long-storage	[75]
Melons, apples, grapes, bananas, lettuce	Postharvest dip	Alkanoyl-L-ascorbic acid esters: 0.075 to 1 mol L^−1^	**Effects:** extension of the shelf life of harvested crops and cost-effective for commercial large-scale manufacturing applications **Drawbacks:** non-natural treatment	[88]
‘McIntosh’ apple	Postharvest spraying	**Stock Solution A:** 0.083 mol L^−1^ Hexanal; 0.057 mol L^−1^ Geraniol; 0.044 mol L^−1^ Geranyl Acetate; 0.030 mol L^−1^ Coumaric acid; 0.0088 mol L^−1^ Benzyl Adenine **Stock Solution B:** 0.056 mol L^−1^ L-Ascorbic acid; 0.024 mol L^−1^ Ascorbyl palmitate; 0.015 mol L^−1^ C-tocopherol; 0.002 mol L^−1^ C-tocopherol acetate **Solution C:** CaCl_2_ 1% (*v*/*v*)	**Effects:** reduction in scald development **Drawbacks:** effects evaluated just in one week, not after long storage like DPA	[66]
‘Braeburn’ apple	Postharvest atmosphere	Harvesting at 3 different stages of maturity: 1 week before optimal harvest date; 1 week after optimal harvest; followed 1-MCP treatment and stored under DCA and CA atmospheres	**Effects:** DCA storage reduced browning disorder compared to CA **Drawbacks:** DCA increases susceptibility to low temperature breakdown and external CO_2_ injury, negative effect that was not observed with DPA application	[89]
‘Starkrimson’ apple	Postharvest atmosphere	Lowest [O_2_] possible	**Effects:** greater tissue resistance to temperature and O_2_-related stresses **Drawbacks:** not as effective as DPA because of core breakdown development	[90]
‘Starkrimson’ and ‘Red Star’ apple, and ‘ Dangshan’ pear	Postharvest immersion ~3 min	10 mg L^−1^ of resveratrol; 1 °C	**Effects:** 88% scald reduction with resveratrol **Drawbacks:** 93% inhibition of scald with DPA	[91]
Apples and pears	Postharvest dip	Coating composition: 0.5% to 1.5% (*w*/*v*) chitosan; 0.5 to 1% (*v*/*v*) glycerol; 0.03 to 0.1% (*w*/*v*) methylcellulose; ad 1.25 to 2.5% (*w*/*v*) gelatine	**Effects:** prevention of scald; inhibition of ethylene production **Drawback:** long-term storage not studied to compare to DPA	[92]
‘Cortland’ and ‘Red Delicious’ apple	342 L stainless steel chamber for 24 h	1-MCP at 1.0 µL L^−1^	**Effects:** evaluation of metabolic pathways involved in superficial scald; antioxidant and redox system, phenylpropanoid metabolism, ethylene biosynthesis, allergens, sulfur amino acids containing proteins and program cell death have a direct link to the scald development.	[93]
‘Fuji Suprema’ apple	Postharvest atmosphere	DCA with respiratory quotient 1.5 and 2.0	**Effects:** inhibition of postharvest disorders **Drawbacks:** fermentative products and reduced ethylene production, weaknesses not observed with DPA use	[94]
‘Granny Smith’ apple	Postharvest atmosphere	DCA at 0 °C for 5 d up to 20 weeks + 6 or 10 weeks simulating handling temperature	**Effects:** DCA controlled superficial scald after 6 weeks of shipment **Drawbacks:** development of the disorder after longer shipments period times	[95]
‘Granny Smith’ apple	Postharvest atmosphere	16 weeks in DCA with a 14 d of interruption in regular air at −0.5 °C, 95% RH; Restorage in DCA	**Effects:** repeated DCA treatments can effectively control scald **Drawbacks:** technologically more demanding than DPA application	[96]
‘Granny Smith’ apple	Postharvest temperature conditioning	10 d at 3 °C and ultra-low oxygen 0.2–0.5 kPa	**Effects:** control of fruit scald **Drawbacks:** core breakdown development, off-flavors production, divergent to conventional application of DPA	[97]
‘Ankara’ pear	Postharvest dip ~5 s	Semperfresh 0.5%, 1.0%, 1.5% (*w*/*v*); Johnfresh: used as it was supplied; 0 °C	**Effects:** effectiveness of both coating in color, firmness, ascorbic acid, titratable acidity, soluble solid retention compared to control pears **Drawbacks:** not as effective as DPA after 9 months of storage	[98]
Fruit and vegetables	Postharvest immersion	Wax containing thymol, eugenol and cinnamaldehyde	**Effects:** control of scald by strengthening the system thymol–eugenol–cinnamaldehyde complex **Drawbacks:** non-natural treatment, not as effective as the conventional application of DPA	[99]
‘Rocha’ pear	Postharvest drench	1-MCP at 0.1, 0.5, and 1.0 µL L^−1^	**Effects:** reduction of superficial scald as effective as DPA **Drawbacks:** non-natural treatment; ripening delay by relatively high concentrations dissimilar to DPA effects	[25]
Fresh produce	Postharvest dip	Beeswax or carnauba wax coating composition: an aqueous phase; one wax 5% (*v*/*v*), one fatty acid and basic amino acid or a salt	**Effects:** protection of fresh produce, from cracking, water loss and oxidation **Drawbacks:** long storage effectiveness not evaluated	[100]
Strawberry, green bean, lettuce	Preharvest application	Fluopyram: 50 mg m^2^	**Effects:** extension of shelf life of fruit and vegetables, namely in controlling microorganisms diseases, such as *Rhizopus stolonifera* **Drawbacks:** not presented	[101]
Avocado, tomato, and guavas	Postharvest dip	A coating comprising: 1-MCP 0.300 mg L^−1^ aqueous emulsion of morpholine oleate, carnauba wax, and shellac; 5 °C and 80% to 90% RH	**Effects:** prevention of water loss; thus, maintaining fruit quality and preventing loss of weight **Drawbacks:** not presented	[102]
Tomatoes, oranges, and peppers	Postharvest dip ~3 min	5 and 10 g L^−1^ of leaves from *Guiera senegalensis, Balanites,* and *Parkia biglobosa*	**Effects:** preservative activity *P. biglobosa > G. senegalensis>Balanites;* **Drawbacks:** higher preservative activity only for 26 days, contrary to long protection offer by conventional DPA application	[103]
‘Lingwu Jujube’ fruit	Postharvest dip ~3 min	Chitosan coating 1% (*m/v*) + cinnamon oil 0.10% (*v*/*v*); 4 °C	**Effects:** reduction of weight and sensory quality loss of jujube fruit; **Drawbacks:** reduction of vitamin C and titratable acid content, physiological changes that did not occur with DPA use	[104]
Peach, cherry, apricot, plum and apple	Postharvest immersion ~1–2 min	Extracts of phellodendron bark, giant knotweed rhizome, and magnolia bark (concentration not revealed)	**Effects:** fruit preservation with plant extract **Drawbacks:** not presented	[105]
Sweet cherry fruit	Postharvest spray	Water solutions of CaCl_2_ and NaHCO_3_ 1% (*w*/*v*)	**Effects:** preservative activity to control postharvest diseases **Drawbacks:** not as effective as conventional DPA effect after 9 months	[106]

DPA = diphenylamine; BHT = butylated hydroxytoluene; CA = controlled atmosphere; DCA = dynamic controlled atmosphere; 1-MCP = 1- methylcyclopropene; RH= relative humidity.
